# Inorganic phosphate is a trigger factor for *Microbispora* sp. ATCC-PTA-5024 growth and NAI-107 production

**DOI:** 10.1186/s12934-014-0133-0

**Published:** 2014-10-10

**Authors:** Anna Giardina, Rosa Alduina, Giuseppe Gallo, Paolo Monciardini, Margherita Sosio, Anna Maria Puglia

**Affiliations:** Department of Biological, Chemical and Pharmaceutical Sciences and Technologies, Università degli Studi di Palermo, Viale delle Scienze – Bd. 16, 90128 Palermo, Italy; Naicons S.r.l., Viale Ortles 22/4, 20139 Milan, Italy; KtedoGen S.r.l., Viale Ortles 22/4, 20139 Milan, Italy

**Keywords:** Ribosomal Post-translationally modified Peptides (RiPPs), Phosphate, PhoP-PhoR, Polyphosphate

## Abstract

**Background:**

NAI-107, produced by the actinomycete *Microbispora* sp. ATCC-PTA-5024, is a promising lantibiotic active against Gram-positive bacteria and currently in late preclinical-phase. Lantibiotics (lanthionine-containing antibiotics) are ribosomally synthesized and post-translationally modified peptides (RiPPs), encoded by structural genes as precursor peptides.

The biosynthesis of biologically active compounds is developmentally controlled and it depends upon a variety of environmental stimuli and conditions. Inorganic phosphate (Pi) usually negatively regulates biologically-active molecule production in Actinomycetes, while it has been reported to have a positive control on lantibiotic production in Firmicutes strains. So far, no information is available concerning the Pi effect on lantibiotic biosynthesis in Actinomycetes.

**Results:**

After having developed a suitable defined medium, Pi-limiting conditions were established and confirmed by quantitative analysis of polyphosphate accumulation and of expression of selected Pho regulon genes, involved in the Pi-limitation stress response. Then, the effect of Pi on *Microbispora* growth and NAI-107 biosynthesis was investigated in a defined medium containing increasing Pi amounts. Altogether, our analyses revealed that phosphate is necessary for growth and positively influences both growth and NAI-107 production up to a concentration of 5 mM. Higher Pi concentrations were not found to further stimulate *Microbispora* growth and NAI-107 production.

**Conclusion:**

These results, on one hand, enlarge the knowledge on *Microbispora* physiology, and, on the other one, could be helpful to develop a robust and economically feasible production process of NAI-107 as a drug for human use.

**Electronic supplementary material:**

The online version of this article (doi:10.1186/s12934-014-0133-0) contains supplementary material, which is available to authorized users.

## Background

Lantibiotics (lanthionine-containing antibiotics) are antimicrobial peptides produced by Gram-positive bacteria belonging to the Firmicutes and Actinobacteria phyla. These ribosomally synthesized and post-translationally modified peptides (RiPPs) are encoded by structural genes (generically named *lanA*) as precursor peptides [1]. Before removal of an N-terminal leader peptide and secretion, the precursor peptide undergoes modifications with the formation of *meso*-lanthionine (Lan) or 3-methyllanthionine (Me-Lan) residues. This occurs via the dehydration of serine and threonine residues to dehydroalanine (Dha) and dehydrobutyrine (Dhb) residues, respectively, then cross-linked via a thioether linkage with cysteine residues [1]. All the genes required for lanthipeptide biosynthesis are usually grouped in gene clusters, also containing genes whose products are involved in additional C-terminal modification, pathway-specific regulation, lantibiotic export and cell immunity [2]. The biosynthesis of many lanthipeptides, e.g. nisin [3], actagardine [4] and NAI-107 [5-7], was genetically characterized. NAI-107, also known as microbisporicin or 107891, is a potent and promising lantibiotic produced by *Microbispora* sp. ATCC-PTA-5024 as a complex of related molecules, with the most abundant congeners, A_1_ and A_2,_ differing by the presence of di-hydroxy- or hydroxy-proline at position 14 and containing a halogenated Trp residue [8]. It is active against Gram-positive bacteria, including methicillin-resistant *Staphylococcus aureus* (MRSA), glycopeptide-intermediate *S. aureus* (GISA) and vancomycin-resistant enterococci (VRE). It has shown superior efficacy in animal models of multidrug resistant infections compared with the drugs of last resort, linezolid and vancomycin [9]. It is currently in late preclinical-phase. The *mlb* and *mib* clusters, containing genes encoding the proteins required for NAI-107 biosynthesis in *M.* sp. ATCC-PTA-5024 and *M. corallina* NRRL 30420, respectively [6,10], are essentially identical.

In *M. corallina* NRRL 30420 it was proposed that an unknown signal, possibly nutrient limitation, activates the positive regulator MibR in a growth rate-dependent manner. MibR triggers the expression of the *mibABCDTUV* operon, leading to the precursor peptide biosynthesis (*mibA*), the core peptide modification (*mibBCDV*) and mature peptide proteolysis and export (*mibTU*). The precursor peptide export would cause release of σ^MibX^ through the inactivation of the anti-σ factor MibW. σ^MibX^ controls, in addition to *mibR*, genes to confer immunity to microbisporicin (*mibEF* and *mibQ*) and genes required for tryptophan chlorination (*mibHS*) and proline hydroxylation (*mibO*), resulting in the formation of fully processed and active microbisporicin [7].

The biosynthesis of biologically active compounds is generally elicited as developmental program and physiological response to a variety of environmental stimuli and conditions, such as the nature and/or quantity of carbon, nitrogen and phosphate sources [11,12]. Inorganic phosphate (Pi) usually regulates antibiotic biosynthesis negatively in both producing Actinomycetes [11,13,14] and engineered strains [15]. In Streptomycetes, cellular response to Pi-limitation stress is controlled by PhoR–PhoP Two Component System (TCS), in which PhoR is a membrane sensor kinase and PhoP is a DNA-binding response regulator. Under Pi limitation, phosphorylated PhoP is able to bind to PHO boxes upstream target genes and controls the expression of *pho* regulon genes. PhoP regulates genes coding for the alkaline phosphatase PhoD, phosphate transporter system PstSCAB and polyphosphate kinase (Ppk), which function in the scavenging of Pi, its transport and its storage as cellular polyphosphates [16,17]. Polyphosphates (PPs) are linear Pi polymers containing high-energy phosphoanhydride bonds. In vitro, they are synthesized when the ATP/ADP ratio is high and degraded when this ratio is low [18]. Both enzymatic activities, polyphosphate kinase (Ppk) and nucleoside diphosphate kinase (NDPK), can reside in the same protein, e.g., PPK2 in *Pseudomonas aeruginosa* [19]. In addition, Pi limitation positively regulates the antibiotic biosynthesis by exerting a control on regulatory genes of actinorhodin and undecilprodigiosin biosynthesis [20]. To the best of our knowledge, no information is available concerning the role of Pi on RiPP production in actinomycetes, although it has been reported that Pi positively influences lantibiotic production in *Lactococcus lactis* and *Micrococcus* sp. GO5 [21,22].

In this study, the effect of Pi on growth and NAI-107 production was investigated at biochemical and molecular genetic levels.

## Results and discussion

### Design of a defined medium

Starting from the mineral composition of Maltose-Glutamate (MG) medium [23], already used for model streptomycetes, such as *Streptomyces coelicolor* [24] and *Streptomyces lividans* [25,26], and other actinomycetes, such as *Amycolatopsis balhimycina* [27,28], four media were developed all containing Glucose (20 or 50 g/l) instead of maltose as carbon source and 60 mM Glutamate (GG20 and GG50) or 25 mM ammonium Nitrate (NG20 and NG50) as nitrogen source. Cell dry weight (CDW) and NAI-107 production were monitored every 24 h till 96 h (Additional file 1: Figure S1). NAI-107 production was verified by bioassay (Additional file 1: Figure S1B) and the presence of both NAI-107 congeners was confirmed by LC-MS analysis (data not shown). All media supported *Microbispora* growth, with medium NG-20 yielding both maximum biomass and maximum production of both congeners of NAI-107. Thus, this medium was used as the defined medium in the following experiments.

### Phosphate role on *Microbispora* growth and NAI-107 production

**Figure 1:**
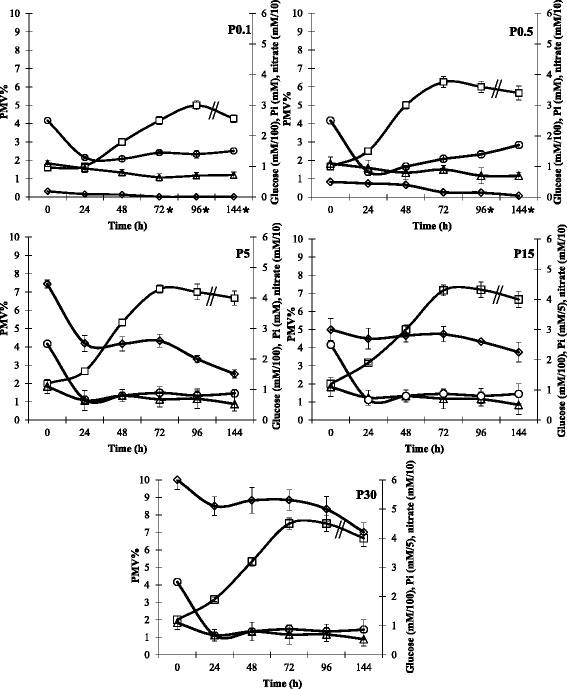
***Microbispora*** **sp. growth in P0.1, P0.5, P5, P15 and P30 media.** PMV% (□), glucose (∆), Pi (**◇**) and nitrate (○) concentrations were monitored. Initial concentrations of glucose and nitrate were 110 and 25 mM, respectively. Initial Pi concentration ranged from 0.1 to 30 mM. Asterisks indicate the growth times where the phosphate concentration was lower than 0.1 mM. Standard deviation was calculated as average of three technical and two biological replicates. Cells were previously inoculated in GE82AB, incubated for 90 h, washed three times, resuspended in distilled sterile water and used to inoculate the different media.

*Microbispora* growth and NAI-107 production were monitored in NG20 medium by varying inorganic phosphate (Pi) concentrations: 0, 0.1, 0.5, 5, 15 and 30 mM (labelled P0, P0.1, P0.5, P5, P15 and P30). *Microbispora* did not grow in P0, indicating that Pi is a prerequisite for growth (data not shown). Glucose, phosphate, nitrate and growth (percent packed mass volume, PMV%) were monitored for the five cultures (Figure 1). Our results indicate that increasing initial Pi concentration improved the cell growth rate (calculated between 24 and 72 h of growth); indeed, cell growth rate was 0.048, 0.072, 0.086 PMV%/h in P0.1, P0.5, P5, respectively. Initial Pi concentration higher than 5 mM did not significantly change the growth rate (0.089 PMV%/h in P15 and P30). Consumption of glucose and nitrate was similar in the five cultures. At the end of the five cultivations, approximately 50–70 mM of glucose and 5–17 mM of nitrate were left, indicating that carbon and nitrogen sources were not limiting for growth (Figure 1). On the contrary, Pi was almost completely depleted (less than 0.1 mM) after 48 h in the P0.1 and after 144 h in the P0.5 (as indicated by asterisks), whereas it was never depleted in the P5, P15 and P30 cultivations.

**Figure 2:**
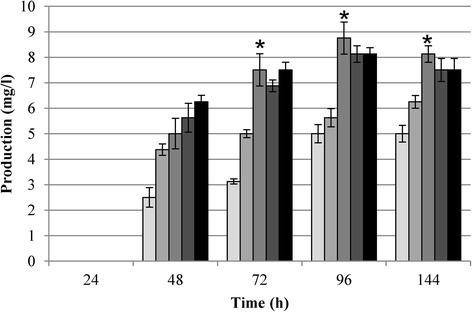
**NAI-107 production in P0.1 (lighter gray bars), P0.5 (light gray bars), P5 (gray bars), P15 (dark gray bars) and P30 (black bars) media.** The NAI-107 presence in the extracts was confirmed by LC-MS. The values were determined with a NAI-107 calibration curve (Additional file 1: Figure S3), as described in MM. Standard deviation was calculated from three independent bioassays. Asterisks indicate the phosphate concentration that allowed the maximum production.

Microbiological assays revealed that NAI-107 was present after 48 h of growth in all cultures (Figure 2). Similarly to biomass accumulation profile, initial Pi concentration from 0.1 to 5 mM stimulated NAI-107 production, and Pi concentrations higher than 5 mM did not further stimulate production (asterisk in Figure 2). In actinomycetes, Pi is known as a key nutritional factor strongly influencing antibiotic production; i.e. biosynthesis of many non ribosomal peptide (NRP) antibiotics is repressed if the cultivation medium contains a Pi concentration higher than 4.2 mM as for A40926 production in batch fermentation of *Nonomuraea* [29,30,13] or 1.8 mM as for balhimycin production in chemostat cultivation of *A. balhimycina* [14]. By contrast, it has been reported that ribosomal biosynthesis of peptides such as nisin, micrococcin GO5, Pep5, epidermin, gallidermin is positively controlled by phosphate; indeed, nisin biosynthesis takes place at high initial phosphate concentrations up to 350 mM and higher [21].

**Figure 3:**
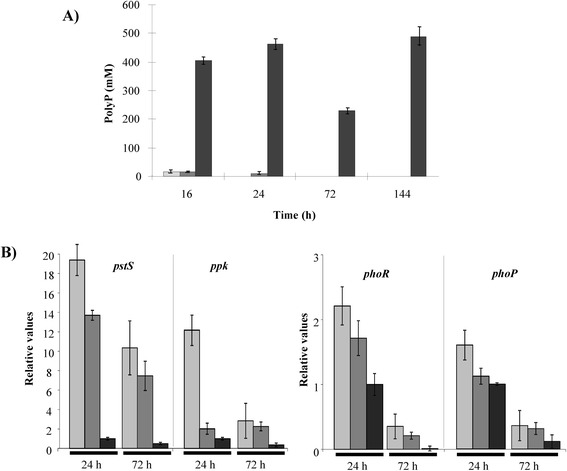
**Pi content of extracted acid-soluble polyphosphates (ASPPs) from** ***Microbispora*** **sp. biomass (A) and transcriptional profile of selected** ***Microbispora*** **sp.** ***pho*** **genes (B) in P0.1 (light gray bars), P0.5 (dark gray bars) and P5 (black bars) media.** Minimum and maximum relative values, calculated from three independent experiments, are reported as error bars. Relative values were normalized using *hrdB* gene as endogenous gene.

To determine that phosphate limitation had occurred, the accumulation of polyphosphates (PPs) and the expression of *pho* genes were analyzed in P0.1 and P0.5 (low phosphate containing media) and compared to those in P5 (one of the high phosphate containing media). PPs are mostly produced in a medium with adequate Pi concentration and are degraded to form ATP in a Pi-limited medium [18] and can be used to monitor if the growth conditions are adequate or limiting of phosphate. As expected, in P0.1 and P0.5 *Microbispora* sp. did not accumulate PPs, while it accumulated PPs already after 16 h of growth in P5 (Figure 3A).

PhoR, PhoP*,* Ppk and PstS encoding genes were identified in the draft sequence of *Microbispora* sp. reported by Sosio et al. [10] by a Blast-P search using the homologues of *Streptosporangium roseum* and *S. coelicolor* A3(2). *Microbispora* PhoR (MPTA5024_26530), PhoP (MPTA5024_26525), Ppk (MPTA5024_37285) and PstS (MPTA5024_31955) show 84% (59%), 98% (84%), 84% (50%) and 80% (50%) sequence identity to the proteins of *Streptosporangium roseum* (or *S. coelicolor*)*.* Sequence comparison indicated that *Microbispora pho* regulon genes have the same organization reported in *S. coelicolor* [17] and contain PHO box direct repeats (DRs) in their upstream region (Additional file 1: Figure S2A). The consensus of *Microbispora* DRs (Additional file 1: Figure S2B), created using free-on line available WebLogo software (http://weblogo.berkeley.edu/logo.cgi), shows high similarity with that of *S. coelicolor* [17]. The pho regulon genes, *pstS* and *ppk*, were previously used as reporters of Pi-limitation in Streptomycetes [18,31-33].

Transcriptional analysis demonstrated that *pstS* and *ppk* gene expression was higher in the P0.1 and P0.5 cultures than in the P5 one (Figure 3B). *phoR* was weakly induced at 24 h (2.2- and 1.8-fold, respectively) and mainly induced at 72 h of growth (27- and 20-fold, respectively) in the P0.1 and P0.5 compared to the P5 culture and *phoP* transcription levels were almost similar in all the tested conditions. Altogether these results revealed that Pi was limiting in both P0.1 and P0.5.

Since in P0.5 medium, *Microbispora* showed a more similar growth rate to P5 than P0.1, P0.5 and P5 were chosen as the low and the high Pi condition, respectively.

### Effect of phosphate concentration on NAI-107 production and *mlb* gene transcription

**Figure 4:**
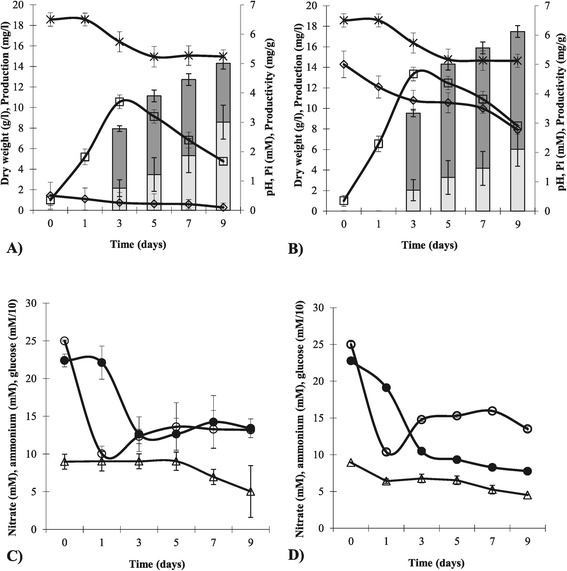
***Microbispora*** **sp. growth in P0.5 and P5 cultures.** Cell dry weigth (white squares), NAI-107 production (dark gray bars), NAI-107 productivity (light gray bars), Pi (white diamonds) and pH (black crosses) of P0.5 **(A)** and P5 **(B)** cultures are shown. The values of production and productivity were standardized with a NAI-107 calibration curve (Additional file 1: Figure S3) as described in MM. Nitrates (white circles), ammonium (black circles) and glucose (white triangles) were monitored during the growth in P0.5 **(C)** and P5 **(D)** media. Initial concentrations of glucose, ammonium and nitrate were 110, 25 and 25 mM, respectively. Standard deviation was calculated as average of three technical and two biological replicates. The NAI-107 presence in the extracts was confirmed by LC-MS.

**Figure 5:**
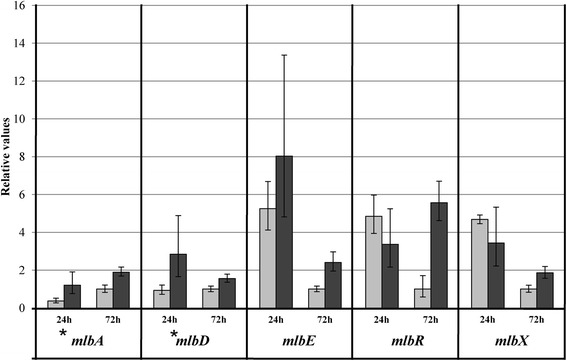
**Quantitative RT-PCR of selected** ***mlb*** **genes of** ***Microbispora*** **sp. after 24 and 72 h of incubation in P0.5 (light gray bars) and P5 (dark gray bars) media.** Minimum and maximum relative values, calculated from three independent experiments, are reported as error bars. Relative values were normalized using *hrdB* gene as endogenous gene. Asterisks indicate genes slightly induced by Pi after 24 h of growth.

To further confirm Pi effect on NAI-107 production, *Microbispora* was grown in P0.5 and P5, as representative conditions of low and high Pi. Biomass dry weight, nitrate, ammonium, pH, glucose and phosphate were determined after 24 h and every 48 h over 9 days (Figure 4). As already demonstrated by Figure 1, the cell growth rate slightly increased in P5 (0.142 g/l*h) in respect to P0.5 (0.111 g/l*h) medium. The quantification of nitrate and ammonium showed that during the biomass accumulation phase (3 days of growth), both nitrogen sources were used (Figure 4C-D). Moreover, both carbon and nitrogen sources were not limiting for growth in both cultivations. At the end of the fermentation, phosphate was almost completely depleted in P0.5 cultivation, while a 2.8 mM concentration was measured in P5 condition. Bioassays of spent medium from *Microbispora* cultures showed that NAI-107 production was detectable after three days of incubation in both P0.5 and P5 media, increased over the entire culture period and it was approximately 1.2-fold greater in the P5 than in the P0.5 medium (Figure 4A-B). It is noteworthy that productivity, calculated as a production/biomass ratio, was not influenced by Pi (Figure 4A-B), indicating that the difference in amount of lantibiotic produced in P0.5 and P5 medium was related to the difference in biomass yield. To evaluate Pi effect on transcription of *mlb* genes, RNAs were extracted after 24 and 72 h of growth in P0.5 and P5 media and analyzed by Quantitative RT-PCR (Figure 5). The transcription levels of two regulatory genes, *mlbR* and *mlbX*, the structural gene, *mlbA*, and genes coding for a modification enzyme, *mlbD*, and for an ABC-transporter, *mlbE*, were studied. As shown in Figure 5, Pi was found to activate transcription (3-fold) only of *mlbA* and *mlbD* at 24 h (indicated by asterisks), while it positively influenced the transcription of all the analyzed genes at 72 h (from 1.5-fold of *mlbD* to 5.6-fold of *mlbR* transcription). The high error bar of *mlbE* transcript level could be due to a high heterogeneity in the expression of this gene at 24 h.

Thus, in accordance with several reports concerning the positive effect of Pi on the biosynthesis of other ribosomal post-translationally peptides in Firmicutes (i.e. nisin*,* micrococcin GO5, Pep5, epidermin, gallidermin) [21-23], also NAI-107 production is positively Pi-controlled.

## Conclusion

The present study provides experimental evidence for the positive effect of inorganic phosphate on *Microbispora* sp. ATCC-PTA-5024 growth and NAI-107 production*.* NAI-107 is a Ribosomal Post-translationally modified Peptide (RiPP) currently in late preclinical-phase.

In the present study, different inorganic phosphate concentrations ranging from 0 to 30 mM were used for *Microbispora* growth, demonstrating that phosphate is necessary and positively influences both growth (Figure 1) and NAI-107 production (Figure 2) up to a concentration of 5 mM. Higher Pi concentrations (15 and 30 mM) were not found to further stimulate *Microbispora* growth and NAI-107 production. In addition, 0.1 mM and 0.5 mM are limiting Pi concentrations, as demonstrated by analysis of PPs accumulation and of *pho* regulon gene transcription (Figure 3). Bioassay and LC-MS analysis demonstrated that Pi has a positive effect on NAI-107 production (Figures 2 and 4) and transcriptional analysis confirmed that selected *mlb* genes, devoted to NAI-107 biosynthesis, were positively influenced by Pi mainly at 72 h (Figure 5). Our results are in accordance with several reports concerning the positive effect of Pi on the biosynthesis of other ribosomal post-translationally peptides in Firmicutes (i.e. nisin*,* micrococcin GO5, Pep5, epidermin, gallidermin) [21-23].

Detailed studies of the lantibiotic biosynthesis and investigation of the effects of other signals will help to understand the physiology of the producer strain and to develop a robust and economically feasible production process.

## Methods

### Strain and media

#### *Microbispora* sp

ATCC-PTA-5024 was stored at −80°C as frozen mycelium in GE82AB medium containing 20% glycerol at a biomass concentration of 6% w/V [8]. The composition of complex and chemically defined media used in this study is listed in Table 1.

**Table 1:** **Composition of media used for** ***Microbispora*** **fermentations**

**Medium**	**Composition (g/L)**	**pH**	**References**
GE82AB	Maltodextrin (20), Soy flour (15), yeast extract (5), CaCO_3_ (1), Agar (1).	7.3	Monciardini, personal communication
MG	Maltose (20), Glutamate (11.23), MOPS (21), MgSO_4_*7H_2_O (0.2), FeSO_4_*7H_2_O (0.09), CaCl_2_*2 H_2_O (0.001), NaCl (0.001), trace elements (as described for R2YE medium), 15 mM PO_4_ buffer.	6.5	Puglia et al. [24]
GG (20 or 50)	Glucose (20 or 50), Glutamate (11.23), MOPS (21), MgSO_4_*7H_2_O (0.2), FeSO_4_*7H_2_O (0.09), CaCl_2_*2 H_2_O (0.001), NaCl (0.001), trace elements (as described for R2YE medium), 15 mM PO_4_ buffer.	6.5	This study
NG (20 or 50)	Glucose (20 or 50), NH_4_NO_3_ (4), MOPS (21), MgSO_4_*7 H_2_O (0.2), FeSO_4_*7 H_2_O (0.09), CaCl_2_*2 H_2_O (0.001), NaCl (0.001), trace elements (as described for R2YE medium), 15 mM PO_4_ buffer.	6.5	This study
P0, P0.1, P0.5, P5, P15 and P30	As NG20 with 0, 0.1, 0.5, 5, 15 and 30 mM of PO_4_ buffer, respectively.	6.5	This study

### Growth conditions and media

For seed culture preparations, 30 ml of GE82AB medium in a 250-ml baffled flask were inoculated with glycerol stock mycelium (6%) and incubated with shaking (200 rpm) at 30°C for 72 h. The resultant culture was subcultured in 30 ml of GE82AB medium using a 6% inoculum. After 90 h of incubation, the second seed culture (6%) was used for inoculating 150 ml of chemically defined medium in a 1-L baffled flask. The culture was incubated at 30°C on a rotary shaker (200 rpm). When indicated (Figure 1), cells were washed three times with distilled sterile water and resuspended in 150 ml of distilled sterile water prior to inoculate the defined media. Samples for determining growth and production of NAI-107 were withdrawn from the culture at different times. All cultivations were run in duplicate and all the measurements were done in triplicate. Standard deviations were calculated from the average of these values.

Biomass was determined by measuring the cell dry weight (CDW) of mycelial pellet recovered from a 1-ml culture sample after drying the pellet at 65°C for about 24 h. Alternatively, it was determined by measuring the packed biomass volume in percent (PMV%), obtained after centrifugation of a 6-ml culture sample for 10 min at 4000 rcf. Growth rates were calculated from the biomass increase between 24 and 72 h in the growing culture.

### Determination of Glucose, Nitrite, Nitrate, Ammonium, Phosphate and polyphosphate concentration

Glucose, Nitrite and Phosphate concentrations were monitored during growth by electronic FreeStyle Freedom Lite® Blood Glucose Monitoring System (Abbott), Griess Reagent Kit for Nitrite Determination (Invitrogen) and EnzCheck® Phosphate Assay Kit (Invitrogen), respectively.

Polyphosphates (PPs) were extracted by an acid method and determined as in [18]. Nitrate amount was measured as described in [34]. The determination of the ammonium ion concentration was performed by a spectrophotometric method using Nessler’s reagent [35].

### Analysis of antibiotic production

NAI-107 production was monitored by bioassay using the paper disc diffusion method with *Micrococcus luteus* ATCC 9341 [36] as test organism. For bioassay, 100 μL of spent medium or extracts were used. The extracts were prepared by adding two volumes of methanol/acetic acid (92/8 based on vol/vol) to 1 mL of culture and incubating at 50°C with shaking for 15 min. The samples were then centrifuged (3000 rpm for 10 min), and the supernatants were applied to Whatman 3 MM Chr paper discs (Wathman, Maidstone, UK). After drying, wet discs were placed on the surface of LB soft agar inoculated with 100 μL *M. luteus* (OD600 = 1.2). Inhibition zones were measured after over night (O.N.) incubation at 37°C. A calibration curve using known concentrations of NAI-107 was constructed and used to calculate NAI-107 production (Additional file 1: Figure S3). Thus, productivity was deduced by normalizing the production for CDW. The presence of NAI-107 congeners in the extracts was verified by LC- MS as described in [8].

### Total RNA isolation and qRT-PCR analysis

To perform transcriptional analysis of selected *pho* and *mlb* genes, mycelium was harvested from 6 ml of culture and resuspended in 1 ml P-buffer containing lysozyme (50 mg/ml) and then incubated for 10 min at 37°C. After two cycles of sonications (30 sec ON/20 sec OFF at output control 4 with Vibra cells, Sonics materials), RNA was extracted by using the RNeasy midi kit (Qiagen) according to the manufacturer’s instructions. DNase I (Roche) treatment was performed at 37°C for 1 h, and RNA was precipitated with 2 volumes of ethanol in the presence of 0.1 volume of 3 M sodium acetate. After a washing step with 70% (v/v) ethanol and air drying, the RNA pellet was resuspended in water with RNaseOUT™ Recombinant Ribonuclease Inhibitor (Invitrogen). As control of RNA quality, a RT-PCR with 0.1 μg of total RNA and primer pairs internal to *hrdB*, encoding a vegetative sigma factor, was carried out using the Superscript One-Step RT-PCR kit (Invitrogen) and the conditions indicated by the supplier. PCRs were performed on 0.5 μg of RNA samples using 40 cycles to exclude the presence of genomic DNA. The identity of RT-PCR products was confirmed by sequencing. For qRT-PCR, a two step protocol was used. The high-capacity cDNA archive kit (Applied Biosystems) was used to retro transcribe 2 μg of total extracted RNA. Primer pairs amplifying intragenic regions of the genes analyzed by qRT-PCR were designed with Primer3web version 4.0.0 (http://bioinfo.ut.ee/primer3/) to fulfill the following criteria: length of 20 ± 2 bp, Tm of 60 ± 1°C, GC content superior to 50% and amplicon lengths ranging from 75 to 150 bp (Table 2). Primer specificity was controlled by Blast analysis. Gene expression was analyzed quantitatively by using Applied Biosystems 7300 real-time PCR system (Applied Biosystems) with SYBR® Green PCR Master Mix (Applied Biosystems) in 96-well plates. Five μL of cDNA were added to 20 μL of PCR. Amplification program consisted in an AmpErase® UNG activation at 50°C for 2 min, in a denaturation at 95°C for 10 min followed by 40 cycles at 95°C for 15 s and 60°C for 1 min. Melting curves were performed from 60 to 95°C to validate the specificity of PCR reaction (Table 2). Three independent measurements were performed for each gene and culture condition. The threshold cycle values (CT) were determined with a baseline set automatically. Results were analysed using the comparative critical threshold method (ΔΔCT) in which the amount of target RNA is adjusted to an internal reference. *hrdB* gene, encoding a vegetative sigma factor, did not show significant expression variation in these experiments (Additional file 1: Figure S4) and was used as an internal reference to normalize the results. Expression ratios were expressed as 2ΔΔCT. In each run, four dilutions of cDNA were analyzed to determine the PCR efficiency and negative controls were included. The amplicons obtained with each primer pair were sequenced.

**Table 2:** **Primers used in RT-PCR analysis**

**Primer**	**Forward**	**Reverse**	**Amplicon size (bp)**	**Amplicon dissociation temperature (°C)**
***hrdB***	GATGCTCTTCCTGGACCTGA	CTTGTTGATCACCTCGACCA	193	87.9
***phoP***	GACAGCGAGATCGACAAGGT	AGCAGCTCGAACTCCTTGAG	239	91.6
***phoR***	ACCGTCGAGATAAGCGTCAG	CTTGCTCCATACCGTGACCT	186	90.3
***pstS***	AGAACTCGCAGCTCCAGAAG	TCGTAGGTCACCAGGACGAT	187	89.6
***ppK***	GACGTCACCGAGAACCTCAT	CAGGCGGTAGATCTCGTTGT	159	88.9
***mlbA***	GACTTCCGAGACCGAGGAC	GTGCACAGCGACCAAGCTC	96	86.3
***mlbB***	GCTGGTTCTTCATCCGCTAC	CTCGTACTCGGGGTCGTACT	179	90.6
***mlbD***	AACCCGGTGATGTGGCGCAAGC	CCGGCCGAGGCATGGTCAGTC	148	90.7
***mlbE***	CACGCTGGCGCTACTTCACCG	TCGAGGCGGCGTTGGAGAGCT	129	90
***mlbY***	CGCCCTGCTCGTGTACGCCGT	GAACGCGGCGATCAGCAGTCC	121	89.4

## Additional file

Additional file 1: Figure S1.
*Microbispora* sp. growth (A) and NAI-107 production (B) in NG20 (black line), NG50 (black broken line), GG20 (light gray line) and GG50 (light gray broken line) media. + and – indicate the positive and negative control, respectively. Positive controls are supernatants of *Microbispora* grown in GE82AB and MG. An empty paper disk and a paper disk soaked in the corresponding defined medium were used as negative controls. Cells were previously inoculated in GE82AB, incubated for 90h and directly used to inoculate the different media. **Figure S2.** Putative PHO box sequences found in upstream regions of *Microbispora pho* regulon genes (A). Translational start codons and transcription direction are indicated with bold letters and arrows, respectively. PHO boxes direct repeats (DRs) are underlined. B) Consensus of the direct repeats of 11 nt that forms the *Microbispora* PHO box, obtained using free-on line available WebLogo software http://weblogo. berkeley.edu/logo.cgi. The height of each letter is proportional to the frequency of the base. Error bars are shown at the top of the stacks. **Figure S3.** Calibration curve constructed using known concentrations of NAI-107. **Figure S4.** Absolute Quantitative RT-PCR of *hrdB* using RNA extracted after 24 and 72h of *Microbispora* sp. growth in P0.1 (light gray), P0.5 (dark gray) and P5 (black) media. Error bars were calculated from three independent experiments (A). The values were calculated using a standard curve obtained measuring fluorescence of known amounts (10, 100, 1000, 10000 μg) of c-DNA (B).
