# Supplementary material for: Inorganic phosphate is a trigger factor for Microbispora sp. ATCC-PTA-5024 growth and NAI-107 production
Source: Microb Cell Fact. 2014 Oct 10;13:133. doi: 10.1186/s12934-014-0133-0 (PMC4203916; doi:10.1186/s12934-014-0133-0)
Supplement: Additional file 1: Figure S1. — Microbispora sp. growth (A) and NAI-107 production (B) in NG20 (black line), NG50 (black broken line), GG20 (light gray line) and GG50 (light gray broken line) media. + and – indicate the positive and negative control, respectively. Positive controls are supernatants of Microbispora grown in GE82AB and MG. An empty paper disk and a paper disk soaked in the corresponding defined medium were used as negative controls. Cells were previously inoculated in GE82AB, incubated for 90h and directly used to inoculate the different media. Figure S2. Putative PHO box sequences found in upstream regions of Microbispora pho regulon genes (A). Translational start codons and transcription direction are indicated with bold letters and arrows, respectively. PHO boxes direct repeats (DRs) are underlined. B) Consensus of the direct repeats of 11 nt that forms the Microbispora PHO box, obtained using free-on line available WebLogo software http://weblogo. berkeley.edu/logo.cgi. The height of each letter is proportional to the frequency of the base. Error bars are shown at the top of the stacks. Figure S3. Calibration curve constructed using known concentrations of NAI-107. Figure S4. Absolute Quantitative RT-PCR of hrdB using RNA extracted after 24 and 72h of Microbispora sp. growth in P0.1 (light gray), P0.5 (dark gray) and P5 (black) media. Error bars were calculated from three independent experiments (A). The values were calculated using a standard curve obtained measuring fluorescence of known amounts (10, 100, 1000, 10000 μg) of c-DNA (B). [file 12934_2014_133_MOESM1_ESM.ppt]

## Slide 1
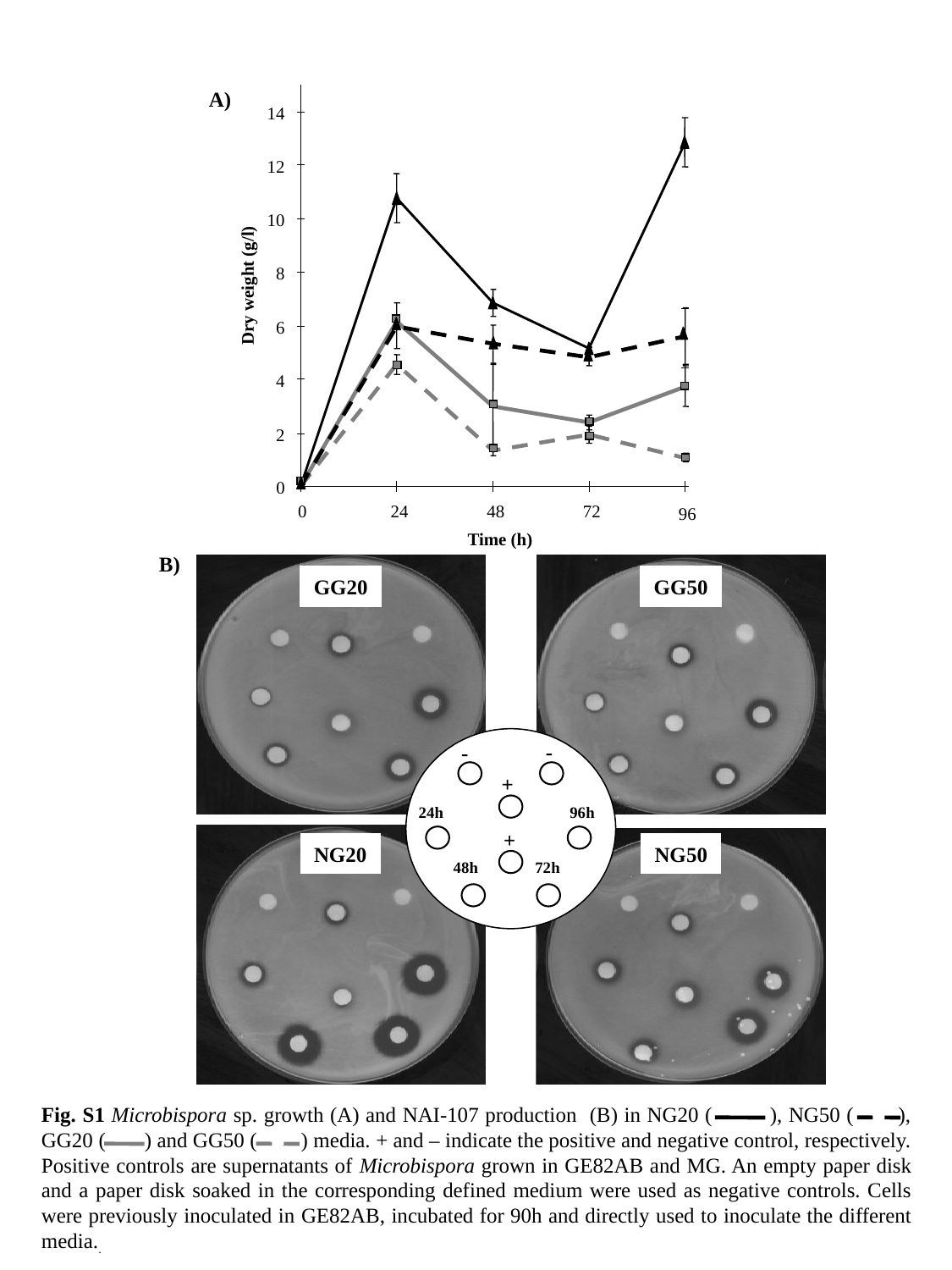

A)
14
12
10
8
Dry weight (g/l)
6
4
2
0
0
24
48
72
96
Time (h)
B)
GG20
NG20
GG50
NG50
-
-
-
+
24h
96h
+
48h
72h
Fig. S1 Microbispora sp. growth (A) and NAI-107 production (B) in NG20 ( ), NG50 ( ), GG20 ( ) and GG50 ( ) media. + and – indicate the positive and negative control, respectively. Positive controls are supernatants of Microbispora grown in GE82AB and MG. An empty paper disk and a paper disk soaked in the corresponding defined medium were used as negative controls. Cells were previously inoculated in GE82AB, incubated for 90h and directly used to inoculate the different media..

## Slide 2
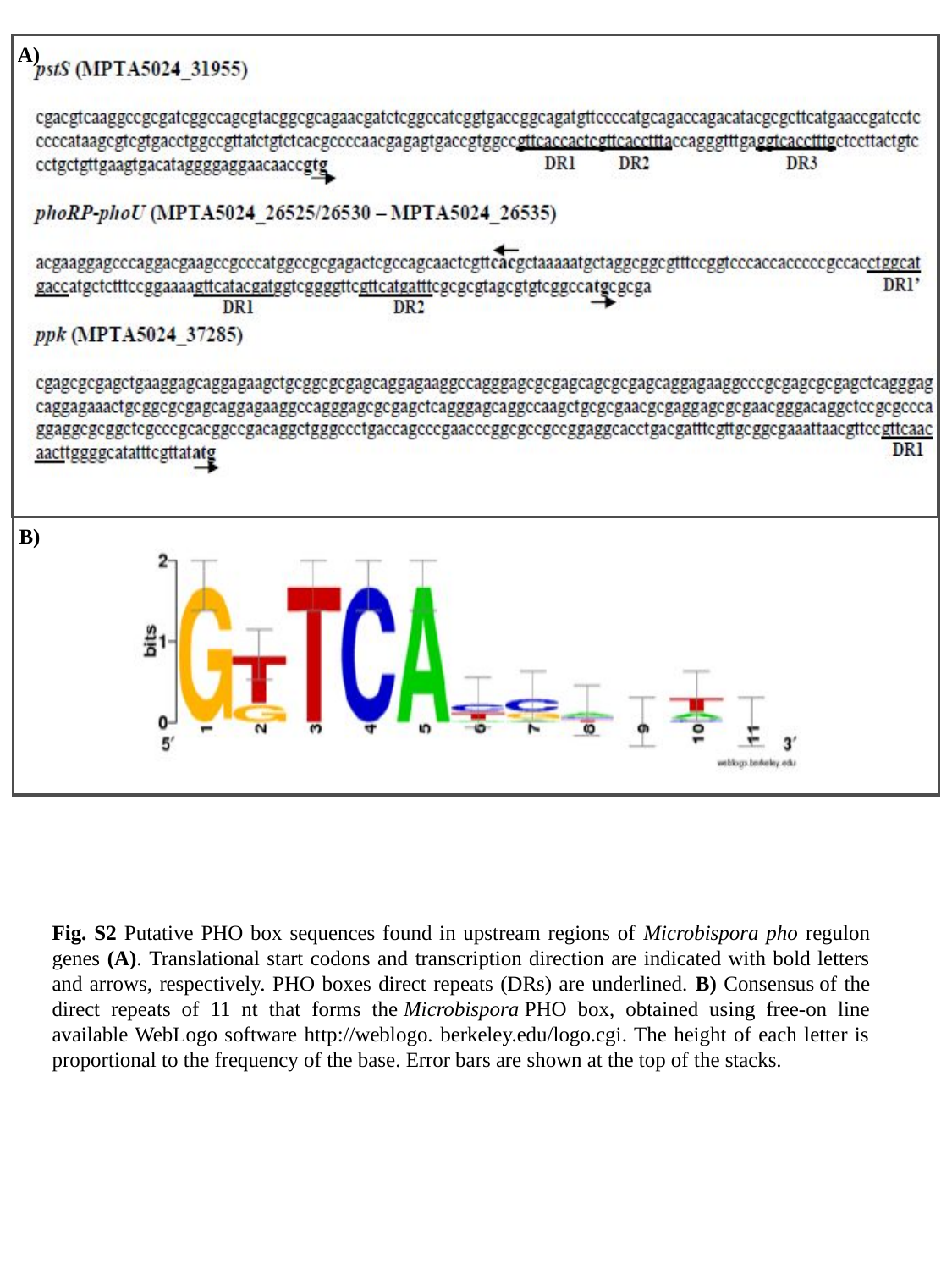

A)
B)
Fig. S2 Putative PHO box sequences found in upstream regions of Microbispora pho regulon genes (A). Translational start codons and transcription direction are indicated with bold letters and arrows, respectively. PHO boxes direct repeats (DRs) are underlined. B) Consensus of the direct repeats of 11 nt that forms the Microbispora PHO box, obtained using free-on line available WebLogo software http://weblogo. berkeley.edu/logo.cgi. The height of each letter is proportional to the frequency of the base. Error bars are shown at the top of the stacks.

## Slide 3
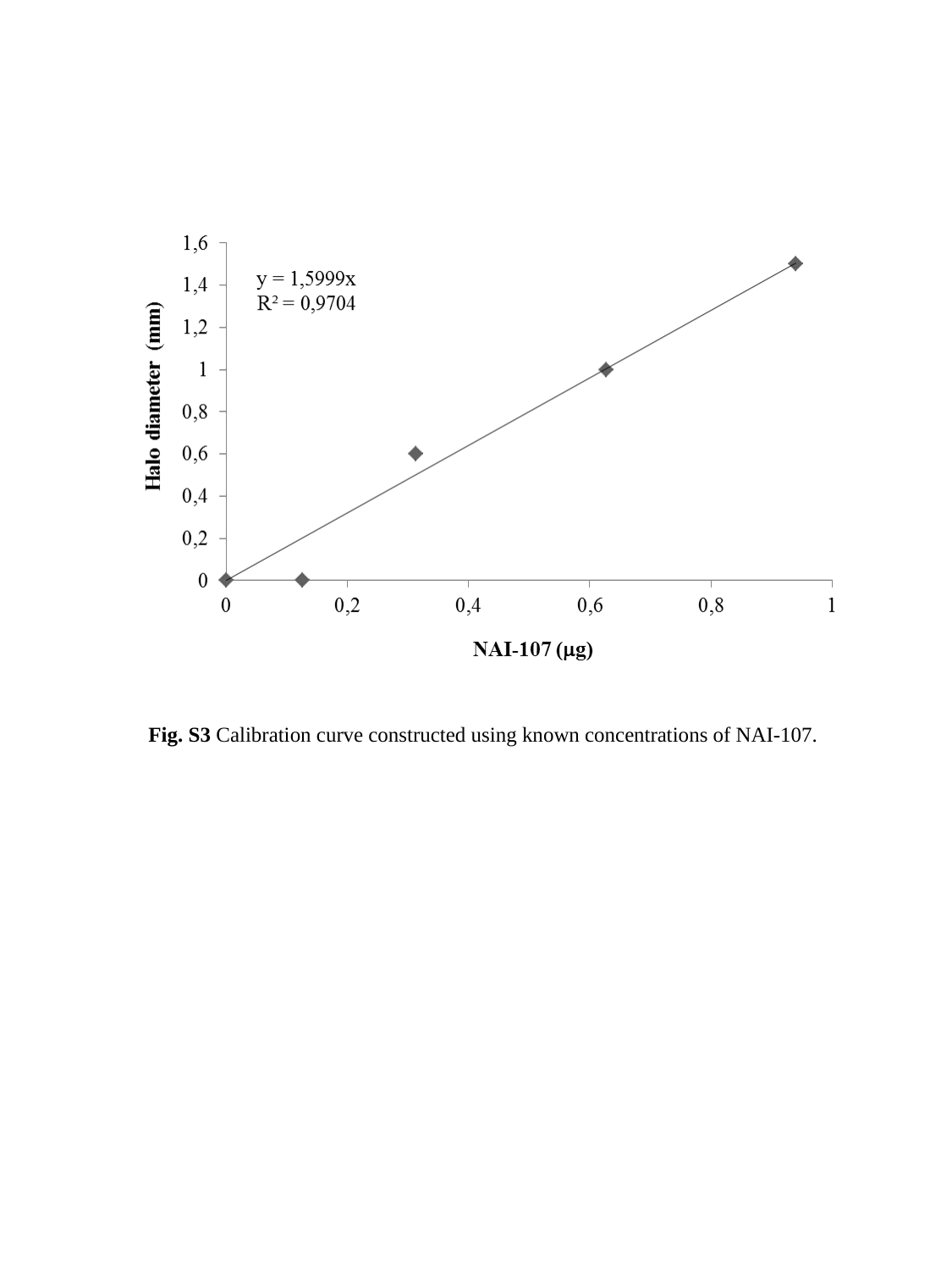

Fig. S3 Calibration curve constructed using known concentrations of NAI-107.

## Slide 4
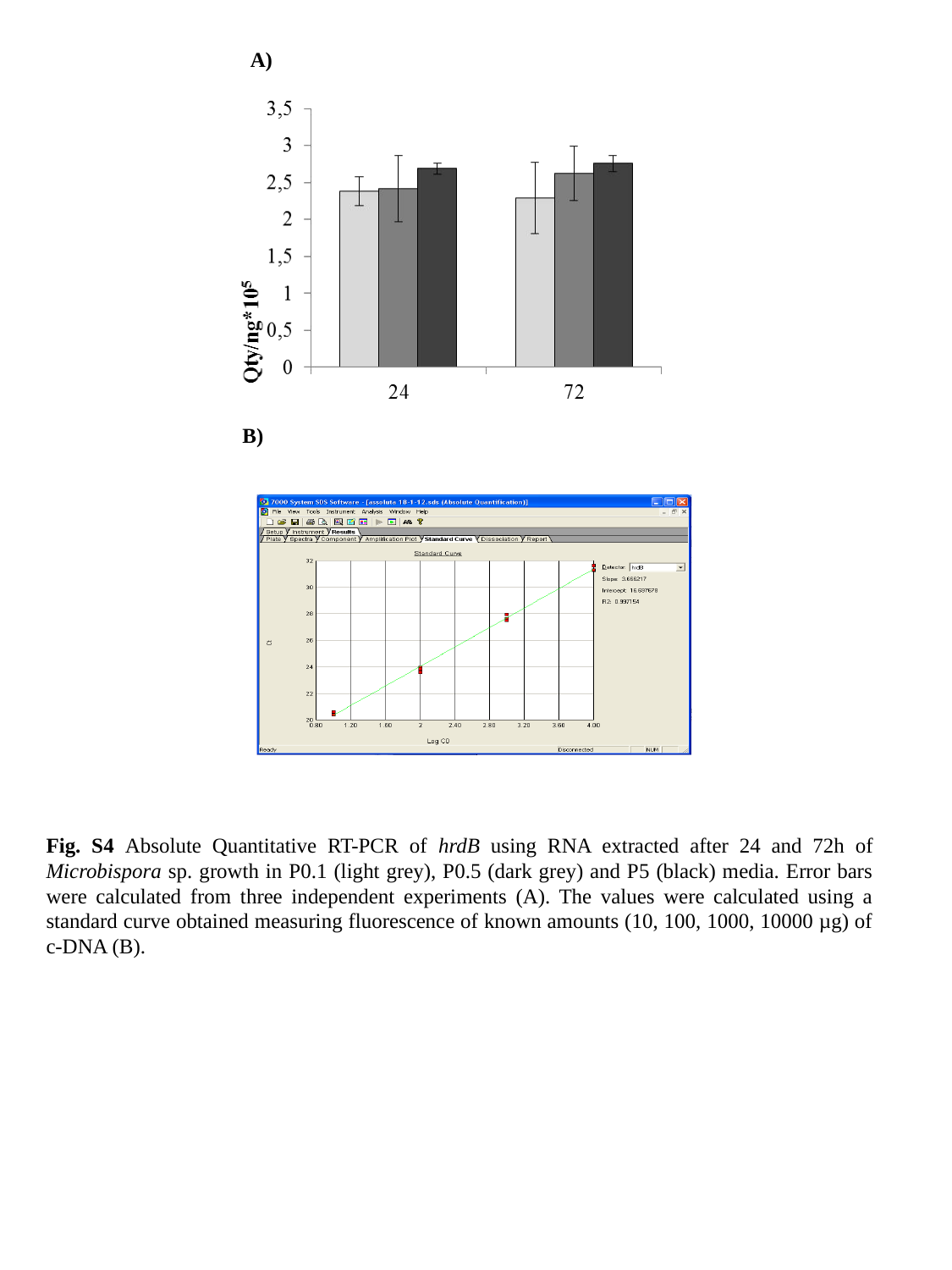

A)
B)
Fig. S4 Absolute Quantitative RT-PCR of hrdB using RNA extracted after 24 and 72h of Microbispora sp. growth in P0.1 (light grey), P0.5 (dark grey) and P5 (black) media. Error bars were calculated from three independent experiments (A). The values were calculated using a standard curve obtained measuring fluorescence of known amounts (10, 100, 1000, 10000 µg) of c-DNA (B).
